# Decrease in Attentional Performance After Repeated Bouts of High Intensity Exercise in Association-Football Referees and Assistant Referees

**DOI:** 10.3389/fpsyg.2019.02014

**Published:** 2019-09-06

**Authors:** Sergio L. Schmidt, Guilherme J. Schmidt, Catarina S. Padilla, Eunice N. Simões, Julio C. Tolentino, Paulo R. Barroso, Jorge H. Narciso, Erik S. Godoy, Rubens L. Costa Filho

**Affiliations:** ^1^Federal University of the State of Rio de Janeiro, Rio de Janeiro, Brazil; ^2^Pontifical Catholic University of Rio de Janeiro, Rio de Janeiro, Brazil; ^3^Federation of Football-Association of the State of Rio de Janeiro, Rio de Janeiro, Brazil

**Keywords:** neuropsychology, attention, sports, performance, cognition

## Abstract

Referees and assistant referees are submitted to high physical stress during matches. Pressure to make decisions in front of large crowds is another potential stressor. These two stressors can impair attention executive control, depending on physical fitness and individual vulnerability or resilience to situational pressure. Error percentage for referees and assistants may reach around 14% during a soccer match. Although previous studies have suggested that soccer referees and assistants should take cognitive assessments, they are only required by Fédération Internationale de Football Association (FIFA) to demonstrate knowledge of the rules and pass annually in a fitness test (FIFA-Test). This study aimed to assess attention performance in referees and assistants before and after the mandatory FIFA-Test. It is hypothesized that the high physical demands associated with the pressure to pass the FIFA-Test would interfere with attention performance. The sample included 33 referees and 20 assistants. The Continuous Visual Attention Test (CVAT) consisted of a 15-min Go/No-go task. Performance in the CVAT is based on four variables: omission and commission errors, reaction time, and variability of reaction time (VRT). Failure in the CVAT was defined by a performance below the 5th percentile of the age- and sex-matched normative data in at least one variable of the CVAT. Before the FIFA-Test all participants performed the CVAT. The second CVAT began 3–7 min directly following completion of the FIFA-test. Considering only the officials who passed both the FIFA-Test and the first CVAT (19 referees and 15 assistants), 44% (9 referees and 6 assistants) exhibited a performance decline in the second CVAT. A significant increase in VRT was found after the high intensity exercise. As increase in VRT is thought to reflect executive dysfunctions and lapses of attention, we concluded that physical fitness alone may not be enough to help officials cope with the physical and contextual stresses associated with the FIFA-Test. These data suggest that over 35% of soccer referees and their assistants who were considered physically able to referee matches may not be mentally prepared for the attentional demands of refereeing soccer matches.

## Introduction

Association football or soccer is played by 250 million players in over 200 countries. The Fédération Internationale de Football Association (FIFA) is the international governing body of association football. A soccer referees’ decisions, as in other sports, can influence the result (Lane et al., 2006; MacMahon et al., 2007). Match analyses carried out during the FIFA Confederations Cup 2009 showed that the error percentage for the referees was around 14% (Mallo et al., 2012). Previous investigators showed that assistant referees make errors on 20–26% of offside calls (Oudejans et al., 2005; Helsen et al., 2006; Catteeuw et al., 2010; Hüttermann et al., 2017). One possible explanation for this high error rate would be the physical demands associated with a soccer match. Another explanation could be the rapid rate of play.

Because of the progress in physical preparation of athletes, soccer players have improved their physical performance (Rampinini et al., 2007). Consequently, referees and their assistants are subjected to an intense physical demand (Lamonte and Ainsworth, 2001; Castagna et al., 2007) without substitution during the matches, except in case of an injury. During a competitive match, a soccer referee may cover nearly 11 km (4–18% at high intensity), attaining approximately 90% of maximal heart rate and 80% of maximal oxygen uptake (Barros et al., 2007).

Exercise is considered a stressor (Tomporowski and Ellis, 1986). “Stress” may be defined as a state of threatened homeostasis, which is counteracted by adaptive processes to regain homeostasis (McEwen, 2007). Thus, exercise will induce increased peripheral and central catecholamine concentrations (McCorry, 2007; Arnsten, 2009). Furthermore, in response to an acute bout of exercise the adrenal gland is stimulated to release cortisol (Goncharova, 2013).

As nor-epinephrine and dopamine strongly influence cognition, one would expect improved cognitive functioning during and after the physical stress induced by exercise. However, when physical stress levels are high, nor-epinephrine and dopamine concentrations become excessive. This high excess of nor-epinephrine activates lower affinity adrenoreceptors which results in reduced neuronal firing in the prefrontal cortex (PFC) impairing attention and executive control (Arnsten, 2009). The PFC is also involved in motor function during high intensity exercise workout (Robertson and Marino, 2016). This can lead to increased demand in the PFC. As described for the catecholamines, the level of cortisol must be neither too high nor too low, to obtain maximum cognitive improvements (Goncharova, 2013). In this way, several studies have reported that moderate physical exercise improves cognitive performance while high intensity exercise does not (Isaacs and Pohlman, 1991; McMorris and Keen, 1994; Covassin et al., 2007; Lo Bue-Estes et al., 2008; Moore R.D. et al., 2012; Wohlwend et al., 2017). In contrast, however, athletes are capable of maintaining their cognitive performance also during high physical exercise (Hüttermann and Memmert, 2014). The meta-analysis by Chang et al. (2012) suggested that the negative effect of high-intense exercise on cognitive performance was only found in studies that included participants with low fitness levels. Accordingly, Labelle et al. (2013) showed that the cognitive performance of lower fit individuals was worse than higher fit subjects during high intensity exercises.

The optimal exercise intensity to improve cognitive function remains controversial (Kashihara et al., 2009; Lambourne and Tomporowski, 2010; Davranche et al., 2015). Part of the controversy may be explained by the absence of uniformity regarding the control of crucial variables that can influence cognitive performance after and during physical activity such as exercise intensity and duration, physical fitness, physical task complexity, type of exercise (continuous or intermittent), and the task used to assess cognitive performance.

Referring a soccer match superimposes a high intensity exercise onto a high cognitive demand. A high cognitive demand may be exemplified by the number of decisions that a soccer referee makes during a match (MacMahon et al., 2007), which may cause mental exertion. In this regard, it has been proposed that prolonged mental exertion decreases visual attention and executive functions (Boksem et al., 2005; Hallet, 2007; Faber et al., 2012). Besides the impact of mental exertion on cognitive performance, it should be mentioned that the concept of fatigue includes the effects of exercise on both muscles and brain. Peripheral fatigue has been defined as an inability to maintain muscle power or force (Barnett, 2006). The manifestations of peripheral fatigue in the brain include altered decision-making and decrease in attention performance (Rattray et al., 2015). Therefore, most fatigue associated with physical activity involves both peripheral and central nervous system fatigue (Lambert and Flynn, 2002; Zaja̧c et al., 2015). In addition, the pressure to make decisions in short times as well as players’ reaction and crowd interaction can be viewed as situational stressors. Psychological stress consists of stimuli that threaten the individual’s current or anticipated state (Herman et al., 2016). It has been suggested that the brain uses response pathways that vary according to the two different kinds of stressors (Sawchenko et al., 2000; Hill et al., 2008). Taken together acute stress is thought to impair pre-frontal attention functions through the excessive release of nor-epinephrine and cortisol (Ulrich-Lai and Herman, 2009). Therefore, either physiological stress from exercise or psychological stressors may impair attentional control leading to distractibility and difficulties in sustaining attention (Sänger et al., 2014).

As referees and assistants are subjected to physical and psychological stressors at the same time, a previous investigation suggested that executive attention performance should be used for screening referees and assistants (Pietraszewski et al., 2014). However, decisions about referees’ qualifications for officiating at games take into account only their knowledge of the rules and their physical condition. In this regard, soccer referees and assistants are required to take a physical test annually. This mandatory physical test (FIFA-Test) is regulated by FIFA and is used in all countries in which soccer is a professional game (FIFA, 2010).

The FIFA-Test has been created to reflect the physical demands during a match. The mandatory FIFA-Test can also be considered a situational stress, because the participants are pressured to pass the fitness test. In addition, the high physical performance that is required to complete the FIFA-Test successfully may also interfere with the referees’ cognitive performance. Therefore, it would be of interest to study the cognitive performance of soccer referees and assistants before, during, and after the FIFA-Test. To accomplish this study at least two issues must be addressed: the moment of the cognitive assessment and the task used for the cognitive evaluation.

The moment the cognitive assessment is done – before, during, or after the exercise may influence the performance. Previous studies examining the immediate effects of exercise on cognition, while participants are engaged in physical activity, have reported contradictory results (Wrisberg and Herbert, 1976; Brisswalter et al., 1995; Audiffren et al., 2008; Moore R.D. et al., 2012; Perini et al., 2016). After an acute bout of exercise, heart rate recovery was usually achieved after 1 to 2 min (Shetler et al., 2001; Kannankeril et al., 2004; Pierpont and Voth, 2004). In this study the cognitive task was administered from 3 to 7 min directly following completion of the FIFA-Test. The issue of administrating the second attention task at 3–7 min after the FIFA-Test will be fully considered in the section “Discussion” after reporting the results.

The task used for the cognitive assessments also plays a crucial role in studies concerning the effect of acute exercise on cognition (Padulo et al., 2016). The hierarchical organization of human cognition points out that the attention domain occupies a top hierarchical position (Lezak, 1995). Therefore, one can consider that deficits in different domains may be secondary to a primary attention problem. The present study focuses on assessments of attention performance after acute exercise. However, attention itself consists of different subdomains. Petersen and Posner (2012) identified several different attention networks that operate, at least in part, independently. The networks include subdomains such as alerting, orienting, executive control, and self-control. Llorens et al. (2015b) analyzed the effect of acute exercise on covert and overt attention. It should be mentioned that the attention subdomains described by Petersen and Posner (2012) could directly be assessed by the Attentional Network Task (Fan et al., 2005). However, test–retest reliability studies with this instrument were not available in Brazil. The present study objectively assessed the alerting and the executive attention subdomains with the aid of a continuous performance test (CPT) validated and normalized in Brazil. Thus, the subdomains of attention described here derived entirely from studies using visual CPTs. In this regard, Egeland and Kovalik-Gran (2010) found correlations between the CPT variables and the attention subdomains. More recently, Schmidt et al. (2016), Simões et al. (2017), and Simões et al. (2018a) have reported that attention–deficit–disorder (ADHD) patients exhibit particular deficits on CPT variables. Simões et al. (2018b) further demonstrated that these deficits were associated with different attention subdomains. According to these studies, impaired performance on CPTs could be explained by the following four conditions: (1) a drop in vigilance caused by falling activation which causes slow reaction times (RTs) (alertness subdomain); (2) occasional lapses in attention as test progresses, affecting the stability of response times which causes an increase in the variability of the RTs (VRTs) (executive-sustained-attention subdomain); (3) failure of focused attention, severe enough to result in errors of omission (focused attention subdomain); and (4) inability to control inadequate responses (impulsivity subdomain) resulting in a high number of commission errors (CEs). This study objectively assessed the alerting and the executive attention subdomains with the aid of a CPT validated and normalized in Brazil.

In Brazil, the Continuous Visual Attention Test (CVAT) is commonly used for attentional assessments (Schmidt et al., 2008, 2016, 2017; Avellar et al., 2016; Simões et al., 2017; Simões et al., 2018a, b). The CVAT is a typical Go/No-go task approved for clinical use in Brazil that does not depend on IQ, has robust internal consistency and construct validity, as well as a high test–retest reliability (Schmidt and Manhães, 2004). The CVAT gives four parameters to assess attentional performance. The standardized scores of the four parameters of the CVAT were based on more than 1000 subjects, which incorporated subjects from different cities around the country. Test–retest reliability studies showed that there is no learning effect in the event of a retest (Schmidt and Manhães, 2004). Here, the performance of each participant was compared to age- and sex normative data of the four parameters. Poor performance on the CVAT was defined as scoring below the 5th percentile in one or more parameters of the test. Thus, a normal performance on the test indicated that the subject did not differ from the general population in each one of the four parameters of the CVAT at a 5% level.

This study aimed to assess attentional performance in soccer referees and assistants before and after the mandatory FIFA-Test. The following specific objectives and respective hypotheses were addressed:

First objective: To describe the attentional pattern of all participants in the FIFA-Test immediately before the physical test (baseline performance) compared to the expected values of the general population based on age- and sex-matched normative data. We hypothesized that the attention performance of the participants would be affected by the situational stress associated with the mandatory FIFA-Test.

Second objective: To compare the attentional performance at rest (before exercising) between those who passed the FIFA-Test and those who did not. We hypothesized that high aerobic fitness as demonstrated by passing the FIFA-Test would be correlated to better baseline attentional processing scores on the CVAT.

Third objective: To describe attentional performance after the FIFA-Test in those participants who passed the FIFA-Test and exhibited normal performance in the baseline attention task (at rest, immediately before the FIFA-Test). If good physical fitness, as evaluated by the FIFA-Test, is the condition necessary and sufficient for adequate cognitive performance of referees and their assistants, then one would expect that most of the participants who passed both the baseline attention test (at rest) and the FIFA-Test (after high intensity exercise) will not exhibit a performance decrease in the second attention test after the FIFA-Test. In contrast, if the FIFA-Test is necessary but not sufficient, one would hypothesize that some of the participants who passed both in the attention test at rest and the FIFA-Test will show a performance decline in the second attention test performed after they experience physical and psychological stresses.

This study was carried out in cooperation with the Federation of Association-Football of the State of Rio de Janeiro (FERJ) and the Department of Neurology of the Federal University of the State of Rio de Janeiro, Brazil.

## Materials and Methods

### Participants

Fifty-three Brazilian-native participants (33 referees and 20 assistant referees) participated in the study. Exclusion criteria included smoking, hypertension, abnormal electrocardiogram, abnormal echocardiography, diabetes, and use of any medication 3 months before the test. Although the officials were not assessed for Attention Deficit Hyperactive Disorder (ADHD), the CVAT has an accuracy >70% to discriminate ADHD patients from healthy controls (Simões et al., 2017). All participants had a minimum of 2 years officiating at professional soccer matches (average number of matches: 25 per year). This study received approval from the local Ethics Committee (Veiga de Almeida Committee – Brazil Federal Platform number: CAEE 006805512.9.0000.5291). According to the law, written informed consent was obtained from all participants.

### Procedures

**FIGURE 1:**
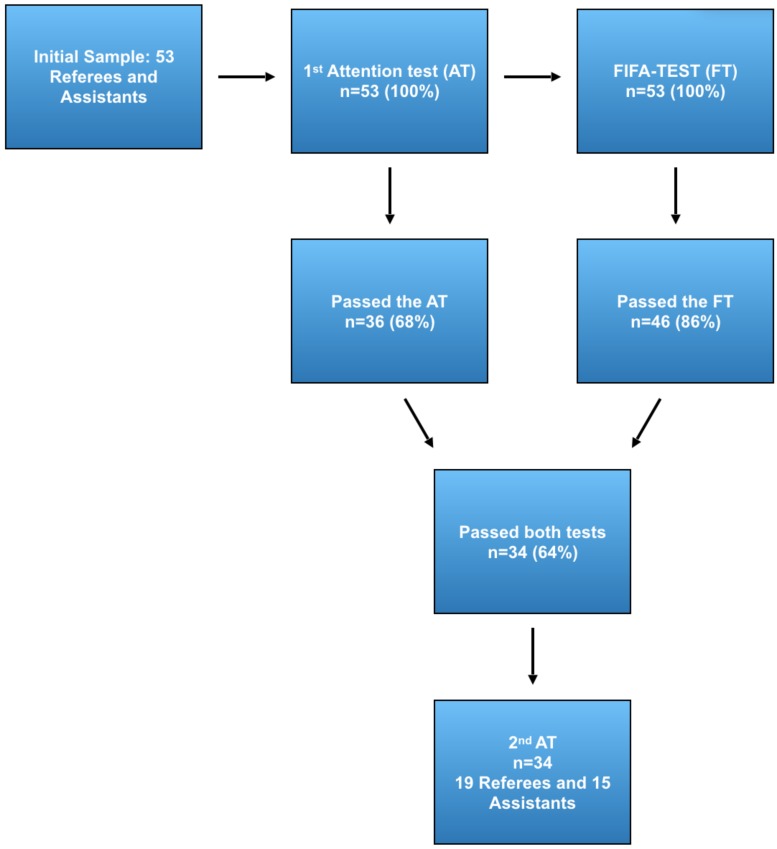
Procedures. Fifty-three referees and assistants participated in the study. Before the FIFA-test all participants performed the first attention task. Those who succeed both in the FIFA-test (*n* = 46) and the first attentional test (*n* = 36) were submitted to the second attention test (19 referees and 15 assistant referees).

All the procedures (Figure 1) were done at the Newton Santos Olympic Stadium in Rio de Janeiro, during the 2017 official physical evaluation of soccer referees and assistants (FIFA-Test). Before the FIFA-Test all participants performed the attention task (first attention test).

When the FIFA-Test ended, the official instructor gave verbal instructions to remind the participants who passed the test to start the second attention task. Then, approved referees and assistant referees received 3–7 min to recovery from the end of the FIFA-Test to the start of the second attention test. During their recovery, the selected participants walked back to the microcomputer where they had performed their first attention task.

### Attention Task (CVAT)

**FIGURE 2:**
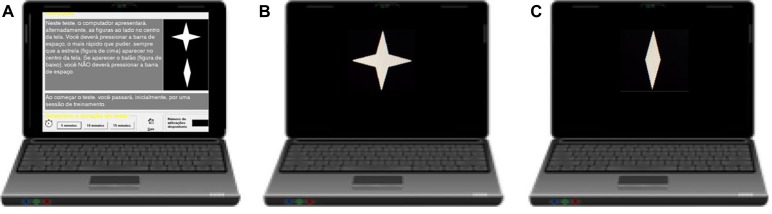
Attention task (CVAT). Computer visual attention test (CVAT). The test begins with written instructions on the screen **(A)**. The target **(B)** remains on the screen for 250 ms. The non-target **(C)** remains on the screen for 250 ms. Inter-stimulus time interval varies between 1, 2, and 4 s, equally distributed along the test. The test lasts 15 min. Translated version of instructions in English: “In this test, the computer alternately displays the indicated figures in the center of the screen. You must press the spacebar using your dominant hand as fast as you can whenever the star appears in the center of the screen. If the other figure appears, you should not press the space bar.”

The testing equipment consisted of a laptop computer linked to a 13-inch liquid–crystal display (Operating System: Windows^®^ 10, maximum time error allowed: 30 ms). The computers were positioned in the lateral part of the soccer field, on the right side near the position where the participants finished their physical tests. Participants were seated in front of the computer in such a way as to allow the hands to be placed over the keyboard. The distance between the center of the monitor and the eyes was approximately 50 cm. Before each task, the examiner instructed the subject to press the spacebar on the keyboard as fast as possible each time a specific visual target stimulus was displayed on the monitor. The test started with instructions and a practice session. The practice sessions took 30 s in both pre and post FIFA-Test. There were six blocks with three sub-blocks each of 20 trials (two figures presented whether targets or not). For each block, the sub-blocks had different interstimulus time intervals (ISI): 1, 2, or 4 s. The order of the ISIs varied between blocks. Half of the sequences had 80% target probability, and the other half had 20% target probability. These target probabilities were randomly assigned during the test. Each stimulus was displayed for 250 ms. There were 180 targets and 180 non-targets and the total test took 15 min to complete. The types of measures included omission errors (OEs), CEs, RTs, and VRT (Figure 2). As the FIFA-Test started at 6 PM, all participants performed the attention tasks at the same time of the day.

### The FIFA Fitness Test

Referees and assistants referee must pass the FIFA-Test at least once a year. The FIFA fitness testing was conducted by two qualified physical instructors and four auxiliaries. An equipped ambulance and a physician were always present during the entire testing sessions. A minimum of three researches and two neuropsychologists were always presented during the sessions. The testing sessions took place in four different days and were always started at 6 PM. For each day, the FIFA-Test was done in groups consisting of no more than 16 referees or assistant referees.

The official fitness test for referees consisted of two subtests:

First subtest (Repeated Sprint Ability): It measures the referees’ ability to perform six repeated sprints over 40 m (maximum time allowed: 6 s per trial); Referees received 60 s recovery between each of the 6 × 40 m sprints. During their recovery, referees walked back to the start. If a referee failed one trial out of the six, they were given a seventh trial immediately after the sixth trial. If a referee failed two trials out of seven, the match official instructor informed that this participant failed the test.

Second subtest (Interval Test): It evaluates the referees capacity to perform a series of high-speed runs over 75 m (maximum time allowed = 15 s) interspersed with 25 m walking intervals (recovery time = 20 s). Total number of runs was 40; minimum speed for the high-speed runs was 18 km/h. Referees completed 40 × 75 m run + 25 m walk intervals, a total of 4000 m. At the end of each run, each referee must enter the walking area before the command given by the official test instructor (whistle). If a referee failed to place a foot inside the walking area on time, he received a warning from the test instructor. If a referee failed to place a foot inside the walking area on time for a second occasion, he was stopped by the test instructor and informed that he had failed the test.

Fédération Internationale de Football Association states that the time between the end of first subtest and the start of the second should be 6–8 min maximum. Here, the time between the end of first subtest and the start of second subtest was set to 8 min. The FIFA-Test for the referees took 38 min to complete. The referees had to complete the two subtests.

The official fitness test for assistant referees consisted of three subtests:

First subtest (CODA): It assesses the assistant referee’s ability to change direction; Assistant referees sprint 10 m forward, 8 m sideways left, 8 m sideways right, and 10 m forward. If an assistant referee failed, he or she was given an additional trial. If he or she failed two trials, the test official instructor informed that the participant had failed the test. Reference time for men assistant referees: maximum 10 s per trial. Reference time for women assistant referees: maximum 11 s per trial.

Second subtest (Repeated Sprint Ability): It measures the assistant referee’s ability to perform five sprints over 30 m (maximum time allowed per trial = 4.70 s for men and 5.10 s for women). Assistant referees had 30 s recovery between each of the 5 × 30 m sprints. During their recovery, assistant referees had to walk back to the start point. If an assistant referee failed, he or she was given an extra trial immediately after the failed sprint. If the participant failed two trials out of six, the test instructor informed he or she had failed the test.

Third subtest (Interval Test): It evaluates the assistant referee’s capacity to perform a series of high-speed runs over 75 m (time allowed = 15 s, per trial) interspersed with 25 m walking intervals (recovery time = 22 s per trial). Total number of speed-runs was 40 and the minimum speed was 18 km/h.

The time between the end of first subtest and the start of second subtest was set to 4 min. The time between the end of second subtest and the start of third subtest was set to 8 min. The FIFA-Test for the assistant referees took 40 min to complete. In order to pass the FIFA-Test the assistants had to complete all the three subtests.

### Statistical Analysis

First objective: The attentional performance of each subject in the total sample (*n* = 53) was compared with the normative values for same age and gender (normalization sample which includes more than one thousand subjects). A subject was considered to fail in the attention test if he or she exhibited a significant deficit (below the fifth percentile in at least one of the four parameters of the CVAT). Therefore, the percentage of subjects in the general population that could be considered as showing an abnormal attention performance was estimated as a maximum of 5% for each one of the four parameters. Consequently, in the most conservative scenario, the maximum expected probability of finding an abnormal performance on one variable of the CVAT would be 20% (5% × 4) in the general population. The *Z*-test for proportions was used to verify whether the participants differ from the 20% probability expected in the general population.

Second objective: Then, the total sample (*n* = 53) was divided into two groups based on the performance on the FIFA-Test: pass or fail. The chi-square test was used to study the relationship between physical fitness (pass or fail on the FIFA-Test) and presence of an abnormal attention performance (yes or no) at the baseline compared to the general population. Fisher exact test was also performed due to the sample size.

Third objective: For the following analyses, the selected sample included only participants who had passed both the FIFA-Test and the baseline attentional test. As the FIFA-Test is different for referees and assistants, the analyses were performed separately for each one of the categories (assistants or referees). Repeated MANOVAs were conducted to test if there were a significant change in performance of the CVAT over time (after and before the FIFA-Test) among referees or assistants who did not fail both in the physical test and in the first attention test. Within-subjects factor: TIME of the FIFA-Test (high intensity exercise effect on attention after the FIFA-Test compared to the baseline). Dependent variables: OEs, CEs, RT, and VRT. Then, univariate tests were conducted to verify the effect of high intensity exercise on attentional performance (before and after the FIFA-Test). For each CVAT variable, pairwise *t*-tests were performed comparing attentional performance between the two attentional tests (before and after). The null hypothesis was tested against the hypothesis of a decline in attentional performance after the FIFA-Test. For the assistant referees, gender was used as a covariate. Considering the possible role of non-balanced individual differences, we also conducted *t*-tests using the following index for each subject: [(T2 − T1)/(T1 + T2)] × 100, where T2 was the performance in the second test, and T1 the performance in the first test. This ratio corrected a possible scale effect due to the total performance. This index corrects for any possible baseline differences. Thus, the differences between the groups after and before the FIFA-Test could be directly compared in percentage terms corrected by the performance at the baseline (before the treatment). A positive index indicates that performance after the FIFA-Test is worse than before. T-tests were conducted to test the hypothesis of absence of difference between the two groups.

For all analyses: Type 1 error (α) was set at 0.05, one-tailed, for all comparisons. Corrections for multiple comparisons were performed using Benjamini–Hochberg procedure (Benjamini and Hochberg, 1995). For each one of the differences on the dependent variables, Cohen’s *d* was used to calculate the effect size of the results. For each one of the ANOVAS, η^2^ (eta-squared) was computed. SPSS Statistics for Windows, version 21.0 (SPSS Inc., Chicago, IL, United States) was used for the statistical analyses.

### Sample Size

The formula for the sample size (Np) required to compare pairwise difference is:

$$\text{Np}={\left[\frac{\left(Z\,\alpha /2+Z\,\beta \right).\sigma }{D}\right]}^{2},$$

where:

α = Type I error; for α = 0.05, *Z*α/2 = 1.96,

β = Type II error; for β = 0.20, *Z*β = 0.84. β level was set at 0.20 and power (which = 1–β) was 0.80,

σ = common standard deviation (based on a previous test–retest reliability study with 200 subjects),

*D* = minimum difference accepted, as indicated above.

For the calculation of the sample size, we considered the minimum differences (*D*). The values of these differences were estimated considering that they must reach magnitude levels that had clinical significance. Based on these assumptions and the age and gender (normative data) of the participants, we considered for OEs, *D* = 5 errors; CEs, *D* = 8 errors; RT, *D* = 80 ms, and VRT, *D* = 30 ms. The common standard deviation (σ) was based on a previous test–retest reliability study with 200 subjects.

The analyses for each parameter of the CVAT showed that a minimum of six subjects was required for the pairwise comparisons (after vs. before).

## Results

Initially, 53 participants (33 referees and 20 assistant referees) completed the first attention test (CVAT) before the FIFA-Test (Table 1). From the total initial sample (*n* = 53), 34 participants (19 referees and 15 assistant referees) (Table 2) passed both the first attention test and the FIFA-Test. All the 34 participants were able to start the second attention test during the allowed recovery time (3–7 min).

**TABLE 1:** Anthropometric measures (total sample).

	**Referees (*n* = 33)**	**Male assistant referees (*n* = 10)**	**Female assistant referees (*n* = 10)**
Age (years) ± *SD*	29.2 ± 3.8	24.5 ± 2.4	29.3 ± 4.0
Height (m) ± *SD*	1.79 ± 0.73	1.74 ± 0.60	1.66 ± 0.55
Weight (kg) ± *SD*	76.8 ± 4.8	70.6 ± 4.3	60.5 ± 3.9
BMI (kg/m^2^) ± *SD*	23.2 ± 1.6	22.8 ± 2.0	22.0 ± 1.5

*SD, standard deviation; BMI, body mass index. Values are mean ± *SD*.*

**TABLE 2:** Anthropometric measures (those who accomplished both the FIFA-Test and the first attentional test at rest).

	**Referees (*n* = 19)**	**Male assistant referees (*n* = 7)**	**Female assistant referees (*n* = 8)**
Age (years) ± *SD*	27.8 ± 4.7	23.9 ± 2.2	28.9 ± 3.4
Height (m) ± *SD*	1.80 ± 0.70	1.73 ± 0.54	1.61 ± 0.48
Weight (kg) ± *SD*	77.3 ± 3.5	70.2 ± 4.1	59.2 ± 4.5
BMI (kg/m^2^) ± *SD*	23.4 ± 1.3	23.3 ± 1.1	22.9 ± 1.4

*SD, standard deviation; BMI, body mass index. Values are mean ± *SD*.*

The sample to address the third objective consisted of 19 referees (all men) and 15 assistants (seven males and eight females). Based on the estimated minimum sample size, these sample sizes were good enough to find clinically relevant differences before and after the FIFA-Test.

First objective: Abnormal attention performance at the baseline were found in 32% of the participants (*n* = 17). This high percentage of attention problems exhibited by the participants reached significance compared to the general population (*Z* = 2.18, *P* < 5%). For the general population, the maximum expected percentage of attention problems on at least one CVAT parameter was estimated at 20%, since for each one of the four parameters of the CVAT, the cutoff score was defined by the 5% level.

Second objective: From the 17 participants who failed the first attention test, 5 did not pass the FIFA-Test. As seven participants failed the FIFA-Test, we concluded that in the baseline test (before the FIFA-Test) abnormal attentional performance was found in 71% of the participants who later failed in the FIFA-Test. In contrast, only 26% (*n* = 12) of the participants who did not fail in the FIFA-Test (*n* = 46) exhibited abnormal attention performances in the first test. This difference reached statistical significance (Fisher’s exact test, chi-square = 5.05, *df* = 1, *p* = 0.037). Due to the small sample sizes, Fisher’s correction was applied.

Third objective: Those who passed both the FIFA-Test and the first attentional test were submitted to the second attention test (*n* = 34, 19 referees and 15 assistant referees). Among those that passed both the FIFA-Test and the baseline attention task, 44% (six assistant referees and nine referees) exhibited an abnormal performance in the attention task after the FIFA-Test, based on the expected values of the general population. In addition, the descriptive statistics based on the mean values of the CVAT parameters indicated that the referees (Table 3) and their assistants (Table 4) exhibited a tendential decrease in the executive attention subdomain after the FIFA-Test, except for the number of CEs.

**TABLE 3:** Attentional performance on CVAT – referees before and after the FIFA-Test.

	**Before**			**After**			**Index**
**Parameter**	**Mean ± SEM**	**Min**	**Max**	**Mean ± SEM**	**Min**	**Max**	**Mean ± SEM**
%OE	1.0 ± 0.32	0	6	1.1 ± 0.21	0	3	0.05 ± 0.16 (NS)
%CE	3.37 ± 0.45	1	9	3.32 ± 0.41	0	6	−1.8 ± 1.50 (NS)
RT (ms)	381.05 ± 9.50	336	512	389.53 ± 11.40	338	557	1.05 ± 0.51 (NS)
VRT (ms)	78.84 ± 6.94	45	157	108.26 ± 11.75	50	239	13.0 ± 4.80 ^∗^*p* ; 0.05

*CVAT, Continuous Visual Attention Test; SEM, standard error of the mean; %OE, percentage of omission errors; %CE, percentage of commission errors; RT, reaction time; VRT, variability of reaction time. NS, *p*-value not significant; Min, minimum value; Max, Maximum value; Index = (T2 − T1/T1 + T2) ^∗^ 100, where T1 = pre FIFA-Test performance and T2 = post FIFA-Test performance.*

**TABLE 4:** Attentional performance on CVAT – assistant referees before and after the FIFA-test.

	**Before**			**After**			**Index**
**Parameter**	**Mean ± SEM**	**Min**	**Max**	**Mean ± SEM**	**Min**	**Max**	**Mean ± SEM**
%OE	0.73 ± 0.25	0	3	2.47 ± 0.59	0	6	1.80 ± 0.24 ^∗^*p* < 0.01
%CE	3.27 ± 0.53	0	8	3.07 ± 0.34	1	6	−5.12 ± 4.02 (NS)
RT (ms)	391.20 ± 13.62	316	503	407.20 ± 18.52	310	596	1.67 ± 1.44 (NS)
VRT (ms)	77.40 ± 7.96	36	158	101.87 ± 16.98	37	281	8.89 ± 4.14 ^∗^*p* < 0.05

*CVAT, Continuous Visual Attention Test; SEM, standard error of the mean; %OE, percentage of omission errors; %CE, percentage of commission errors; RT, reaction time; VRT, variability of reaction time. NS, *p*-value not significant; Min, minimum value; Max, maximum value; Index = (T2 − T1/T1 + T2) ^∗^ 100, where T1 = pre FIFA-Test performance and T2 = post FIFA-Test performance.*

**FIGURE 3:**
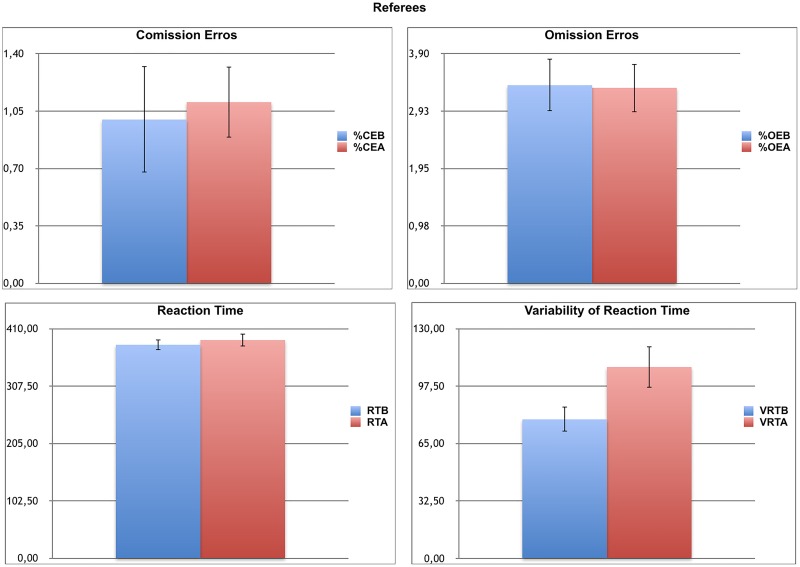
Attentional Performance of Soccer Referees before and after the physical fitness test. Note the significant increase in VRT after the FIFA-Test. %CE, percentage of commission errors; %CEB, percentage of commission errors before the FIFA-Test; %CEA, percentage of commission errors after the FIFA-Test; %OE, percentage of omission errors; %OEB, percentage of omission errors before the FIFA-Test; %OEA, percentage of omission errors after the FIFA-Test; RT, reaction time; RTB, reaction time before the FIFA-Test; RTA, reaction time after the FIFA-Test; VRT, variability of reaction time; VRTB, variability of reaction time before FIFA-Test; VRTA, variability of reaction time after the FIFA-Test; CVAT, Computer Visual Attention Test. Data are expressed as means and each bar is the respective standard error of the mean.

For the referees (Figure 3), repeated MANOVA showed there was a significant effect of the TIME of the FIFA-Test (first vs. second attention test) on the performance of the CVAT over time (*F* = 2.13, *df* = 4/15, *p* = 0.05, η^2^ = 0.39). Considering the significant effect of TIME of the FIFA-Test (before vs. after) on attentional performance identified in the MANOVA, the next set of analyses assessed the effect of the TIME of the FIFA-Test on each one of the four variables of the CVAT. This was done with the aid of univariate ANOVAS. The univariate tests showed a significant effect of the TIME of the FIFA-Test on CVAT for the VRT (*F* = 6.96, *df* = 1/18, *p* = 0.017, η^2^ = 0.26). A tendency for significance was found for RT (*F* = 4.19, *df* = 1/18, *p* = 0.058, η^2^ = 0.15). In contrast, univariate tests indicated there was no effect of TIME of the FIFA-Test on CVAT for OE (*F* = 0.16, *df* = 1/18, *p* = 0.75), and for CE (*F* = 0.01, *df* = 1/18, *p* = 0.89). The significant effect for VRT is explained by the increase in VRT in the second attention test. The same tendency was observed for RT. Subsequent pairwise comparisons confirmed a significant increase in VRT in the second test, after adjusting for multiple comparisons.

**FIGURE 4:**
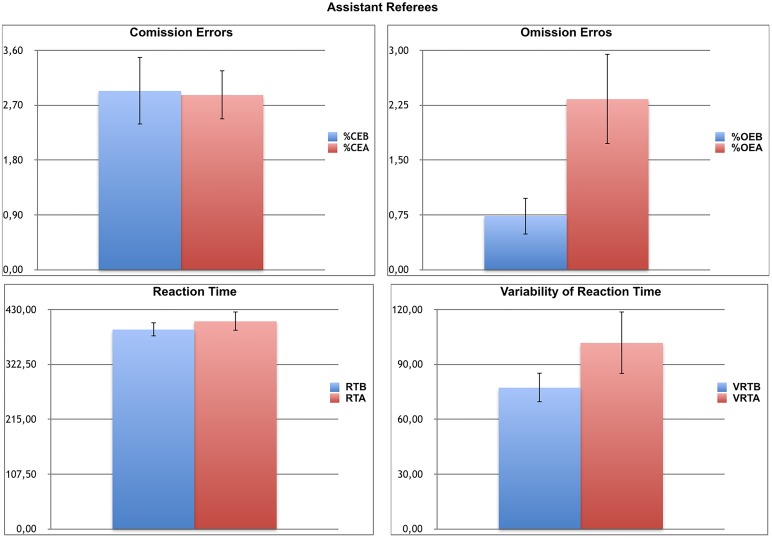
Attentional Performance of Referee Assistants before and after the physical fitness test. Note the significant increase in VRT and %OE in the second attention test. %CE, percentage of commission errors; %CEB, percentage of commission errors before the FIFA-Test; %CEA, percentage of commission errors after the FIFA-Test; %OE, percentage of omission errors; %OEB, percentage of omission errors before the FIFA-Test; %OEA, percentage of omission errors after the FIFA-Test; RT, reaction time; RTB, reaction time before the FIFA-Test; RTA, reaction time after the FIFA-Test; VRT, variability of reaction time; VRTB, variability of reaction time before FIFA-Test; VRTA, variability of reaction time after the FIFA-Test; CVAT, Computer Visual Attention Test. Data are expressed as means and each bar is the respective standard error of the mean.

For the assistants (Figure 4), repeated MANOVA, using gender as a cofactor, showed there was a significant effect of the TIME of the FIFA-Test (first vs. second attention test) on the performance of the CVAT over time (*F* = 3.21, *df* = 4/10, *p* = 0.03, η^2^ = 0.29). Considering the significant effect of TIME of the FIFA-Test (before × after) on attentional performance identified in the MANOVA, the next set of analyses assessed the effect of the TIME of the FIFA-Test on each one of the four variables of the CVAT with the aid of univariate ANOVAS. The univariate tests showed a significant effect of the TIME of the FIFA-Test on CVAT for the VRT (*F* = 5.45, *df* = 1/13, *p* = 0.03, η^2^ = 0.64) and OE (*F* = 14.71, *df* = 1/13, *p* = 0.002). A tendency for significance was found for RT (*F* = 1.90, *df* = 1/13, *p* = 0.08, η^2^ = 0.15). In contrast, there was no effect of TIME of the FIFA-Test on CVAT for CE (*F* = 0.22, *df* = 1/13, *p* = 0.64). The significant effect for VRT and OE is explained by the increase in both variables observed in the second attention test. The same tendency was found for RT. Subsequent pairwise comparisons confirmed a significant increase in VRT and OE in the second test. Although females tended to present higher values for all the CVAT variables, the interaction between gender and TIME of the FIFA-Test did not reach significance.

The analysis of the mean indices (Tables 3, 4) indicated that there was a decrease in the percentage difference in performance for the parameters of the test that reached significant levels (OE for the assistants and VRT for both the referees and assistants). These results showed that the findings based on the ANOVAS did not depend on baseline differences between the groups.

## Discussion

A significant percentage of the participants showed an abnormal performance in the attention task before the FIFA-Test compared to the general population (first objective). A normal-range performance in the baseline attention test was positively associated with passing the FIFA-Test (second objective). The VRT parameter was significantly affected on the second attention test in referees and assistant referees who were approved in the FIFA-Test and did not exhibit an abnormal attention performance at rest (third objective).

At rest, the finding that the percentage of attention problems in officials pre FIFA-Test is higher than the expected ratio in the general population may reflect the contextual stress associated with the pressure to pass the FIFA-Test. As expected, this psychological stressor might be responsible for the higher percentage of participants who failed the first attention test (32%) compared to the maximum percentage of failure expected for general population (20%).

The present results also indicated that a normal baseline attentional performance at rest was positively correlated with the later success in the FIFA-Test. Assuming that the FIFA-Test distinguishes who are in a better physical condition (passed the FIFA-Test) from the ones with worse physical condition (failed the FIFA-Test), this may indicate that the more highly fit approved participants exhibited better cognitive performance at rest than those who failed the FIFA-Test. This finding is supported by previous investigators who reported that the brain-derived neurotrophic factor is greater in people that regularly exercise (Huang et al., 2014; Lee et al., 2014; Szuhany et al., 2015). Furthermore, previous investigations have shown that a high fitness level confers protection against non-physical stressors, mental or psychological (Norris et al., 1992; Gerber and Pühse, 2009; Li and He, 2009). Rimmele et al. (2007) documented significantly lower cortisol and heart rate responses to psychosocial stress in trained men compared with untrained men. In addition, higher physical fitness is associated with lower inflammatory cytokine responses to a mental stressor, as well as greater parasympathetic control, as indicated by less reduction in heart rate variability (Hamer and Steptoe, 2007; Thayer et al., 2012; Ottaviani et al., 2015; Gazzellini et al., 2016). Consequently, this result indicates that physical fitness is a modulator of attention functioning at rest condition in referees and assistant referees who took the FIFA-Test.

Among those who passed both the FIFA-Test and the baseline attention task, 44% exhibited a low performance in the second attention test. Therefore, the finding of a significant decline in attention performance after the work out could not be explained by outliers, since nearly 50% of the sample presented a decline in attention performance in the second attention test. Particularly, after the FIFA-Test, the approved participants showed a significant increase in intraparticipant VRT. Conversely RT did not differ between the two assessments (before vs. after). These findings are supported by previous studies that reported an increased VRT without changes in RT during the execution of sustained attention tasks in several psychopathological conditions such as attention deficit hyperactivity disorder (Gilden and Hancock, 2007; Simões et al., 2017). Increased VRT is thought to reflect disturbances in executive attentional control including lapses in attention, whereas RT is associated to the alertness subdomain (Simões et al., 2017). The compromised neural circuitry related to the executive control may be related to the simultaneous effect of both mental and physical stressors on the PFC (Sänger et al., 2014). Increased VRT has been found in disturbances of the dopaminergic system, executive dysfunctions, and hyperactivation of the midline cortical structures associated with the default mode network (Northoff and Bermpohl, 2004; Gazzellini et al., 2016). The activation of the default mode network is related to lapses of attention during task-oriented activity (Gazzellini et al., 2016). Recently, Buczylowska and Petermann (2018) have described intraindividual variability in executive performance in healthy adults. Taken together, our findings suggest that the executive attention subdomain might be affected after the high intensity exercise. The significant increase in VRT also indicates that part of the officials approved in the FIFA-Test may exhibit lapses of attention under stressful conditions.

The present finding of a decline in attention performance after the FIFA-TEST might be explained by the inverted-U curve hypothesis that has been proposed to explain the relationship between exercise workload and the activation level of the central nervous system (Davey, 1973; Salmela and Ndoye, 1986; Chang et al., 2012; Hüttermann and Memmert, 2014; Davranche et al., 2015; Llorens et al., 2015a; Herman et al., 2016). According to those studies, moderate physical exercise improves cognitive performance, while high intensity exercise does not. However, there are other studies showing that different fitness levels might also have an influence on the presence or absence of an inverted-U curve (Chang et al., 2012). In the present study, considering that all participants who passed the FIFA-Test were regularly involved in physical activities and could be considered as trained participants, the present results do not support the role of physical fitness in the maintenance of cognitive performance immediately after high intensity exercise. It is possible that differences in the cognitive task employed here (executive attention) may differ from other studies, and then explain this discrepancy. Another explanation may be related to the duration of the FIFA-Test, since it is possible that exercise duration may negatively affect cognitive performance (Schmit et al., 2015). Dietrich and Audiffren (2011) proposed that more than the intensity, duration of exercise would be determinant to account for the cognitive effect of exercise. Another point is that the FIFA-Test consists of repeated bouts of high intensity exercise. In this regard, Tsukamoto et al. (2016) suggested that repeated bouts of high intensively exercise decreased lactate accumulation and reduced the putative positive effect of exercise on executive function performance during the post-exercise recovery. Taken together, it is possible that the duration of the FIFA-Test combined with the intermittent nature of the test would lead to a decrement of cognitive processes efficacy.

Whereas reduced hypothalamic–pituitary–adrenal axis (HPA) reactivity to a given stressor has repeatedly been reported in physically fit individuals, the finding of reduced sympathetic reactivity is less consistent (Silverman and Deuster, 2014). Both dampened and augmented catecholamine stress responses have been demonstrated in high- versus low-fit persons during exposure to psychological stressors (Sothmann et al., 1991; Moyna et al., 1999). This raises the question of the individual response to a combination of physical and situational stresses. Recent findings suggest that individual brain network under acute stress controls the cognitive consequences of threat (Kohn et al., 2017). The configuration of different brain networks may be associated with vulnerability or resilience to stress. Stress responses via catecholaminergic pathways may have selective effects on a network of brain regions that include the temporo-parietal and cingulate cortices, amygdala, thalamus, striatum, and the brainstem. Changes in these regions are followed by changes in the executive control network centered in the PFC. Based on circumstances and individual differences in stress reactivity, the response to stress can be a cognitive facilitation or a cognitive disturbance (Hermans et al., 2011; Sandi, 2013).

The effect of psychological stress on cognitive performance seems to depend on the way the participant sees the FIFA-Test. A previous study reported that those that are particularly susceptible to mental stress are also more affected by physical stress (Stults-Kolehmainen and Sinha, 2014). Our findings support that the individual response to the situational stress (FIFA-Test) may affect executive attention control after the physical stress caused by the FIFA-Test. This interpretation is supported by a recent finding on the negative effect of anxiety on cognitive performance during and after a physical exercise (Nibbeling et al., 2014).

During stressful conditions, the HPA axis comes into play leading to the synthesis and increased the release of glucocorticoids (Herman et al., 2012; Nibbeling et al., 2014). Glucocorticoids bind to mineralocorticoids and glucocorticoids receptors around the PFC (Herman et al., 2012). Thus, stress impairs the PFC control of the attention processing and attentional selection switches from a top-down regulation to a bottom-up control by the sensory cortices. The biopsychosocial model of challenge and threat (BPSM) may, at least in part, explains the present results. The BPSM proposes that the way the stress is evaluated by the subject determines the triggering of distinct neuroendocrine and cardiovascular responses (Blascovich et al., 2004). Both challenge and threat increase sympathetic-adrenomedullary activation causing higher cardiac activity, and increased blood flow to the brain and muscles. A threat evaluation of the situation activates the HPA axis releasing a higher amount of cortisol which causes vasoconstriction and decreased blood flow. Therefore, a threat evaluation impairs the top-down control of the attentional process.

Recent studies have shown that challenge and threated evaluations are related to opposite effects on attention performance (Moore L.J. et al., 2012; Vine et al., 2016). Moore et al. (2013) demonstrated that golfers in a threat state displayed inferior performance compared to golfers in a challenge state. During mandatory check-in flight simulators, pilots that evaluated the task as threat exhibited disrupted attentional control (Vine et al., 2015). We hypothesized that the mental stress associated with the physical stress might affect the performance by those participants who believe that the FIFA-Test is a threat and not a challenge. The physical fitness exercised a positive influence on the attentional performance at rest, but the way the participants see the test (challenge or threat) may influence the attentional performance after exercising even in the participants who passed the FIFA-Test.

In the present study, the sample included females only for the assistant referee subgroup. Although the interaction between SEX and TIME did not reach significance, the performance of the female assistants showed a tendency to be worse than that observed in males. Gonadal steroids are considered to be an important physiological modulator of the HPA axis (Handa and Weiser, 2014). In general, testosterone inhibits stress reactivity and estradiol appears to enhance HPA axis responses (Viau and Meaney, 1996) and, consequently, women show greater variability in stress-induced HPA axis activity than men (Viau and Meaney, 1996; McCormick and Mathews, 2007). It is possible that our finding of an absence of interaction might be explained by the lack of statistical power for the interaction effect since the number of females was low.

This study presents some limitations such as: The small sample size used to address the second objective; anxiety levels were not measured; absence of blood lactate and salivary cortisol measurements, because lactate levels would help in the objective evaluation of the physiological response to exertion, whereas the cortisol level would indirectly measure the stress level; self-reports of challenge and threat were not used; lack of physiological data such as heart rate, hydration, VO_2_ max, and body fat; absence of cardiovascular fitness tests, because stronger participants could pass the test with less physical effort (and lower stress) than less fit ones and, consequently, would perform better cognitively.

The recovery time after the exercise (3–7 min) also posed some limitations to the interpretation of the results. The minimum and maximum times were based on physiological and logistical reasons, respectively. We choose to start the second attention assessment giving a minimum recovery time of 3 min because previous studies have demonstrated that parasympathetic re-activation and sympathetic withdrawal take 1–2 min after heavy exercise in subjects with high fitness (Barnett, 2006). Therefore, starting the second attention assessment in a time interval always >2 min allowed all participants the same cardiological conditions during the second assessment. The maximum time length of 7 min was based on logistical reasons because it was the maximum time interval that a healthy adult takes to walk from the endpoint of the fitness test to the location where the attentional assessments took place. However, most participants performed the second attentional test 4 min after the exercise; that is the time they usually took to cover the distance mentioned above by walking. Although this time interval may be associated with peripheral recovery, it seems unlikely it would lead to brain recovery because the latter involves the action of neurotransmitters in central-nervous synapses, which usually takes much more time than the direct peripheral response. Even though post-exercise recovery involves restoration of peripheral fatigue associated with improvements in afferent feedback, the present study focused on how a physical stressor (FIFA-test) affected the brain in a context of a particular state of anxiety (the outcome of the fitness test may affect officials’ professional future). In this regard, little is known on brain fatigue after repeated bouts of high intensity bouts of exercise (Rattray et al., 2015).

However, when the time allowed to start the second attention assessment is summed with the total length of the attentional task (15 min), we could not entirely discard the hypothesis that at least a partial brain recovery from stressors might have happened. Conversely, prolonged mental exertion during the 15-min attentional task might directly affect the brain (Hallet, 2007) and causes manifestations such as altered decision-making, and decreases in responding to correct targets. It should be mentioned that the total time length of the FIFA-Test was 40 min, which is approximately the same time length of the first half of a soccer match. Thus, the time span from the recovery time (3–7 min; average of 4 min) to the end of the second attentional test might be considered as a useful estimator of a putative decline in attentional performance when officials returned to the soccer field for refereeing the second half of the game (the time interval from the first to the second half is 15 min).

The finding that the percentage of officials who did not pass the first attention test is higher than that expected for the average population may be explained for the negative effect on attention caused by the stress associated with the pressure to pass the fitness test. However, we cannot entirely exclude the possibility that some participants were not motivated enough to perform a lengthy attention test before the fitness test. Although a lack of motivation can explain the high number of officials who did not pass the first attentional assessment (32%), it does not explain the higher percentage of officials who failed both the first attentional assessment and the fitness test (71%) compared to those who also failed the first attention assessment but passed the fitness test (26%).

In the present investigation, the putative decline in attentional performance after the high intensity exercise was studied, excluding all the participants who failed either the first attention task or the FIFA-Test (third objective). However, it would be of some interest to allow all participants to perform the second attention assessment regardless of their performance in the first attentional assessment or the FIFA-Test. For logistical reasons, those who did not pass the FIFA-Test were not included, because they were not allowed to continue the physical test or even stay in the stadium after they had failed in any step of the fitness test. Those who passed the FIFA-Test but failed the first attention task (*n* = 12, including referees and assistants) were not included in the analysis for the following reasons: First, as we cannot exclude the possibility that the participants who failed the first attention test did not give full effort to perform the task, it would not be reasonable to include them in the second assessment. Secondly, even if those participants who failed the first attention assessment were sufficiently motivated, we could not exclude a ceiling effect on some parameters of the CVAT test; thus, a drop in the second assessment would not reflect an actual decrease in performance at least on the parameters presenting the plateau and comparisons (before vs. after) in the four parameters of the CVAT would not be allowed. Third, as the calculation of the minimum sample size was based on an estimation of the variance for the average population, a different sample size is expected if all subjects exhibit poor attentional performance, due to a likely increase in the variance in this population. Fourth, even assuming that the variance of the data does not increase in a sample with poor attention performance, the actual number of participants in this study would not allow a separate analysis because we have two different groups (referees and assistants) with a total number of only 12 subjects. Finally, we have found that an abnormal performance in the first attentional assessment indicates a high chance to fail in the FIFA-Test later (second objective). Consequently, the study on the decline in attentional performance after the FIFA-Test could adequately be done using only the sample that had passed both the first attention test and the FIFA-Test. Further investigation is needed to study the accuracy of the CVAT to identify officials who are likely to exhibit attentional decline after bouts of exercise. For this purpose, the use of a larger sample is needed.

Another important limitation of this study was the absence of attentional assessments in a baseline situation without the situational stress associated with the pressure to pass the FIFA-Test. Further investigation is needed to compare attentional assessments in a neutral situation against those on the day of the FIFA-Test.

On the view of these limitations, further studies are needed to fully address the question of attentional declining after bouts of exercise in stressful situations. As the main finding of the present study is the effect on the VRT, it will be possible to assess officials immediately before the start and after each half of real soccer matches. The feasibility of this proposal is supported by the findings that VRT can reliably be measured using time lengths as short as 90 s (Haynes et al., 2017).

We concluded that physical fitness alone may not be enough to help officials cope with the physical and contextual stresses associated with the FIFA-Test. If the FIFA-Test reflects, in a minor scale, the difficulties in a real match, the present data indicate that at least part of well-physically prepared soccer referees and assistant referees who were considered able to referee matches indeed may not be mentally prepared for the attentional demands of a real match. We suggest that executive attentional performance tasks must be included in the FIFA-Test.

## Data Availability

All datasets generated for this study are included in the manuscript and/or the supplementary files.

## Ethics Statement

This study received approval from the local Research Ethics Committee (CAAE 006805512.9.0000.5291) and written informed consent was obtained from all participants.

## Author Contributions

All authors listed have made a substantial, direct and intellectual contribution to the work, and approved it for publication.

## Conflict of Interest Statement

The authors declare that the research was conducted in the absence of any commercial or financial relationships that could be construed as a potential conflict of interest.
